# Supplementary material for: GsMAS1 Encoding a MADS-box Transcription Factor Enhances the Tolerance to Aluminum Stress in Arabidopsis thaliana
Source: Int J Mol Sci. 2020 Mar 15;21(6):2004. doi: 10.3390/ijms21062004 (PMC7139582; doi:10.3390/ijms21062004)
Supplement: Supplementary file 1 [file ijms-21-02004-s001.pdf]

Additional file 1

Sequence information of CDS and amino acid

CDS sequence of *GsMAS1* (726 bp)

CCGGAATTCAGAGGCTCGAGTTTTTCAGCAGATTTCTCCTTCCAAATCACTCCTATAATTTTCAATTCAAAGG
TGGTATTTGTGAAGGAACCCTAAAATATAATTTTTTGACCAAAG**ATGGGGAGAGGTAAGATTGCG**
ATTCGAAGGATCGACAACTCCACTAGCCGGCAAGTGACTTTCTCAAAGAGAAGA
AATGGATTGCTGAAGAAAGCTAGAGAATTATCAATTCTTTGTGATGCTGAAGTTG
GATTGATGGTGTTCTCCAGCACTGGGAAGCTTTATGACTATGCAAGCACAAGCAT
GAAAGCGGTTATTGAACGCTACAACAAGCTAAAAGAGGAAACCCATCACCTCAT
GAATCCGGCTTCAGAAGAGAAGTTTTGGCAGACAGAAGCAGCAAGCTTGAGGCA
GCAGCTTCAGTACTTGCAAGAATGCCACAGGCAATTAATGGGGGAAGAACTTAC
GGGTTTGGGTATTAAAGAACTACAAAATCTGGAAAACCAACTGGAGATGAGTTT
AAAGGGTGTCCGCATGAAAAAGGATCAAATTTTAACTAATGAGATTAAAGAACT
ACGCCAAAAGGGAAATATCATTCATCAAGAAAATGTTGAACTCTATCAAAAGATG
GAGCAGATCCAAAAAGAAAATGCAGAGCTACAAAAGAAGGTTTATGAAGCAAG
GAGTACAAATGAAGAAAATGTGGCATCCAATCCTTCTTACAACGTCAGAAATGGA
TATGATTCACTTGCATCTATCAGTCTCCAGCTAAGTCAGCCACAGTCTCAATACAA
ATACAGTGAACCATCAACCAAAGCAATGAAACTCGGATTGCAGCTGCATTAGCAA
AACTGTTCAGTAATCAGGGAGGTTGTCATTTGTGCCAAGCCAGCCAACAAATCTTTCTAGAAGATCTCCTAC
AATATTCTCAGCTGCCATGGAAAATCGATGTTCTTCTTTTATTCTCTCAAGATTTTCAGGCTGTATATTAAAAC
TTATATTA

Amino acid sequence of GsMAS1 protein (241 aa)

MGRGKIAIRRIDNSTSRQVTFSKRRNGLLKKARELSILCDAEVGLMVFSSTGKLYDYAS
TSMKAVIERYNKLKEETHHLMNPASEEKFWQTEAASLRQQLQYLQECHRQLMGEEL
TGLGIKELQNLENQLEMSLKGVRMKKDQILTNEIKELRQKGNIIHQENVELYQKMEQI
QKENAELQKKVYEARSTNEENVASNPSYNVRNGYDSLASISLQLSQPQSQYKYSEPSTK
AMKLGLQLH*

Additional file 2

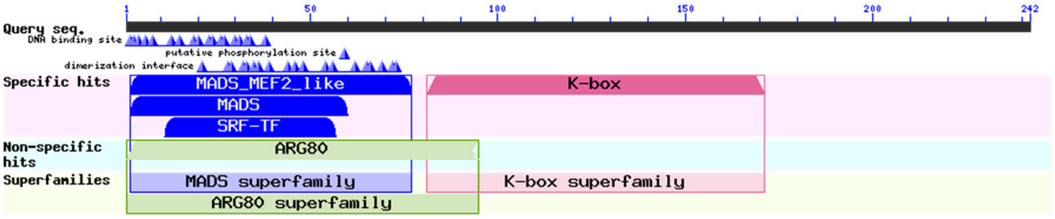

Figure S1 The putative domains of the GsMAS1 protein

The structure diagram comes from the NCBI website of the blast results using amino acid sequence of GsMAS1 protein. It belongs to MEF2 (myocyte enhancer factor 2)-like/Type II subfamily of MADS (MCM1, Agamous, Deficiens), and SRF (serum response factor) box family of eukaryotic transcriptional regulators. The MADS-box domain can bind DNA existing as hetero and homo-dimers.

Additional file 3

Table S1 List of the primers in present study

Serial number	Primer name	Primer sequence (5'to3')	Purpose
1	GsMAS1-F	TTCTCCTTCCAAATCACTC	*GsMAS1* cloning
2	GsMAS1-R	TTGTTGGCTGGCTTGG	
3	pZeroback/Blunt-F	CGACTCACTATAGGGAGAGCGGC	pZeroback-*GsMAS1*
4	pZeroback/Blunt-R	AAGAACATCGATTTTCCATGGCAG	
5	p-GsMAS1-F	gagaacacgggggactctagaATGGGGAGAGGTAAGATTGCG	pTF101.1-*GsMAS1*
6	p-GsMAS1-R	cgatcggggaaattcgagctcCTAATGCAGCTGCAATCCGAG	
7	ACT3-F	GCACCACCGGAGAGAAAATA	qRT-PCR
8	ACT3-R	GTGCACAATTGATGGACCAG	
9	qGsMAS1-F	AGAGAAGTTTTGGCAGACAG	
10	qGsMAS1-R	CCAGTTGGTTTTCCAGATTTTG	
11	At-TUB-F	ATCGATTCCGTTCTCGATGT	
12	At-TUB-R	ATCCAGTTCCTCCTCCCAAC	
13	C-GsMAS1-F	aattctgcagtcgacggtaccAAATGGGGAGAGGTAAGATTGCG	pYL322-d1-eGFP-*GsMAS1*
14	C-GsMAS1R	ccttgctcaccatcaggatccAATGCAGCTGCAATCCGAGTT	
15	PGBKT7- GsMAS1-F	aggacctgcatatggccatggAAATGGGGAGAGGTAAGATTGCG	pGBKT7- GsMAS1
16	PGBKT7- GsMAS1-R	ccgctgcaggtcgacggatccGCTAATGCAGCTGCAATCCGAG	
17	bar-F	CCTTCGCAAGACCCTTCCTC	Molecular identification for *GsMAS1* transgenic lines by bar gene
18	bar-R	CATGCGGACACCCTTTAAAC	
19	PGIP1-F	CAACCTGGTCTATTGACTTATCAAG	Expression patterns of Al stress responsive genes regulated by *GsMAS1* by qRT-PCR
20	PGIP1-R	GAAGAACTGAAGAGGAGCTTCA	
21	PGIP2-F	ATCCCGACTTTTACCGCATC	
22	PGIP2-R	TGCCATTGTGATTCATGTCC	
23	WRKY46-F	ACATCACATCCCCGAAGACG	
24	WRKY46-R	ACTTCTTCGGACTTGGTCGG	
25	ALS3-F	TATCGATCCTTGCCGGGACTTCA	
26	ALS3-R	GCTTGTCTTGGCGTTGCTCCTA	

27	AtMATE-F	CCTTAGCGTTTGTGTTCGATGGAG	
28	AtMATE-R	ACCATGAGTCGATGAGAGGAAGAG	
29	AtALMT1-F	GGCCGACCGTGCTATACGAG	
30	AtALMT1-R	CATGAGTCCTGTGAACTCCC	
31	STOP1-F	TTTCCGCGACTGATGTTTGAT	
32	STOP1-R	ACAGGCATTCGCAATAAGCAT	
33	STOP2-F	GTGGGTTTGCTCTTGTGGGAC	
34	STOP2-R	TGCACGTGTCATTTCTTGAG	
